# Adaptable control policies for variable liquid chromatography columns using deep reinforcement learning

**DOI:** 10.1038/s41598-023-38145-2

**Published:** 2023-07-12

**Authors:** David Andersson, Christoffer Edlund, Brandon Corbett, Rickard Sjögren

**Affiliations:** 1 Sartorius Corporate Research, Umeå, Sweden; 2 Sartorius Corporate Research, Toronto, ON Canada; 3 SynGen AI Technologies AB, Umeå, Sweden; 4 Present Address: V7 Ltd, London, UK; 5 Present Address: ZensAI AB, Umeå, Sweden; 6 Present Address: CellVoyant Technologies Ltd., Bristol, UK

**Keywords:** Engineering, Mathematics and computing, Physics

## Abstract

Controlling chromatography systems for downstream processing of biotherapeutics is challenging because of the highly nonlinear behavior of feed components and complex interactions with binding phases. This challenge is exacerbated by the highly variable binding properties of the chromatography columns. Furthermore, the inability to collect information inside chromatography columns makes real-time control even more problematic. Typical static control policies either perform sub optimally on average owing to column variability or need to be adapted for each column requiring expensive experimentation. Exploiting the recent advances in simulation-based data generation and deep reinforcement learning, we present an adaptable control policy that is learned in a data-driven manner. Our controller learns a control policy by directly manipulating the inlet and outlet flow rates to optimize a reward function that specifies the desired outcome. Training our controller on columns with high variability enables us to create a single policy that adapts to multiple variable columns. Moreover, we show that our learned policy achieves higher productivity, albeit with a somewhat lower purity, than a human-designed benchmark policy. Our study shows that deep reinforcement learning offers a promising route to develop adaptable control policies for more efficient liquid chromatography processing.

## Introduction

In downstream processing of therapeutic proteins, affinity chromatography is the most important among the many separation steps for purifying products to high-quality standards required for medicinal use. The purification process is associated with high costs, partly owing to the large investments made in the initial product development stages but also because of the expensive raw materials and waste arising from the production process. In the case of monoclonal antibodies (mAb), the most common class of biopharmaceuticals, proteins are produced in genetically engineered mammalian cells, and the first purification step is commonly mAb capture using protein-A affinity chromatography. In protein-A affinity chromatography, the specific binding characteristics of mAbs are exploited for separation. The binding can be influenced by changing the pH of the solute by adding salt components. This allows for extraction of the target protein from a mixture of spent growth media. This molecular-scale interaction is subject to immeasurable variations in the makeup of biological components and stochasticity of all possible chemical phenomena that can occur in the system. External input parameters originating in the production chain, coupled with multiple components, make the total product output difficult to forecast. Another common challenge in the capture step is the functional variability in the resin-based columns used for separation. The receptors responsible for binding to the target proteins are embedded onto the surface of the resin beads, which are then packed in cylindrical columns for use in chromatography. Variational effects on performance can be traced to variations in the resin manufacturing as well as column degradation during production. The inherent variability and high cost associated with downstream processing make it challenging, but important, to design optimal control strategies.

The conventional approach to developing control strategies for protein-A chromatography is to use a static recipe derived from heavy experimentation. Relying on a static control process in an inherently variable process risks waste products and incurs significant costs owing to accumulation further downstream in the product line. Coupled with the large cost of the initial product, this challenge incentivizes the development of more adaptable control systems that can optimize the maximal yield in production, even when chromatography columns vary.

In contrast to conventional control systems, more adaptable solutions can be developed using data-driven technologies including machine- and deep learning. Such methodologies are becoming more prevalent in biotechnology, particularly in upstream processing. Downstream processing has seen similar developments with the rise of the digital twin concept, where digital simulation tools have been developed to mimic production hardware^1^. This development is further driven by the ongoing transition to continuous manufacturing using hardware devices such as perfusion cell culture systems, continuous flow reactors, and multi-column chromatography systems^2,3^. The complexities of emerging continuous tooling add significant challenges to bioprocessing, thereby generating a demand for capable predictive solutions. Digital twins using physical models typically require numerical solutions of large systems of equations; however, such operations can be computationally expensive, particularly with regard to fluid dynamics. To model fluid-based separation processes, several chromatography-simulation tools have been proposed in the literature^4–6^. Until recently, mass transfer modeling has been the only option available for the prediction of process performance and control; however, novel methods now allow for the integration of more data-driven procedures. By making use of data-augmented simulation-based training, reinforcement learning (RL) can be used to solve optimization problems in real-time^7^. The approach has been used for complex application control in a wide range of domains^8–10^. In the domain of bioprocessing, a method for optimizing the process flow rate in a cation chromatography setting for the separation of charge variants has been proposed^11^. The method uses fixed policies (time points for loading, washing, and eluting) and varying flow rates with a machine learning method. Our approach suggests the addition of additional control capabilities to a larger system model. This creates a more challenging control problem for a data-driven controller owing to the need for self-generation of control policies. State of the art hardware devices for downstream processing of biopharmaceuticals have the capability for advanced control using variable flow rates and modular routing between columns^12^. However, software has yet to be developed that makes use the large number of possibilities such a system offers.

In this paper, we present the first simulation study demonstrating that RL can be used to develop adaptable control policies for liquid chromatography purification. By training an RL agent to freely control the inlet and outlet flows of a chromatography column to optimize the reward function, we find a policy that achieves better average productivity than a human-design benchmark across a set of variable columns. Our work presents a proof-of-concept for using RL to develop solutions to the problem of optimal control of chromatography columns with inherent variability influencing binding capacity.

## Methods

### Simulation environment

**Figure 1:**
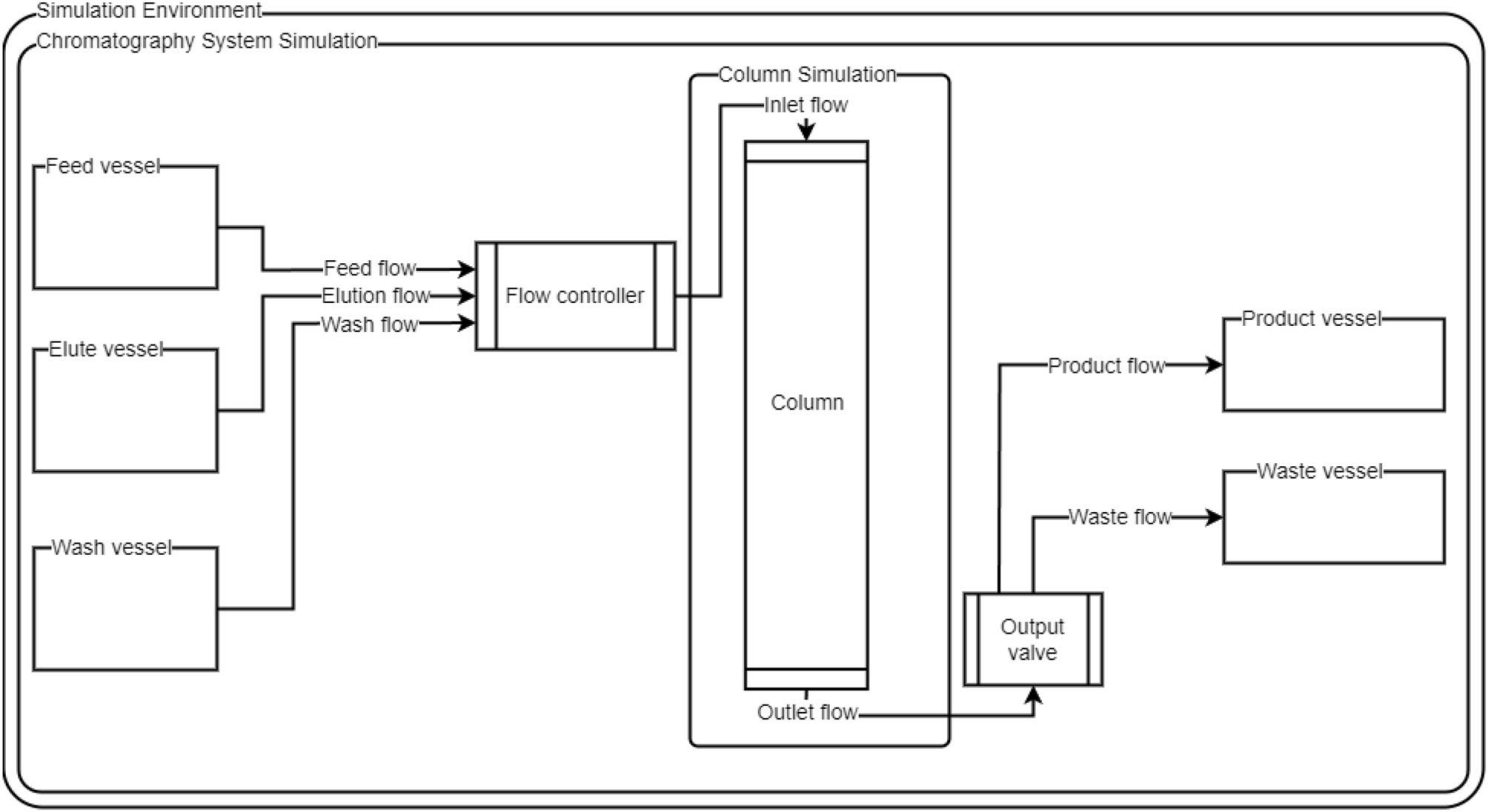
Schematic of the simulation environment containing chromatography system and column simulations. Arrows indicate direction of flow.

A simulation environment describing a single-column bind–elute process (see Fig. 1) was implemented in Python using the Simulation Testbed for Liquid Chromatography software (STLC)^13^. The reinforcement learning environment was implemented using PyTorch and OpenAI Gym^14^. The physical system recreated in the simulation comprises a vessel containing unprocessed media, a vessel containing elution buffer, and a vessel containing wash buffer. Individual volumetric flow rates from the vessels can be set, and their outlets are combined in a common vessel, referred to as the flow controller. The column inlet is fed from the common vessel with combined volumetric flowrates of the individual upstream vessels. At the column outlet, a valve can siphon the processed media into either product collection or waste collection vessels designed to contain purified media and waste.

To represent the physical system, the simulation combines a set of differential equations describing the flow between the various tanks, valves, and columns. The column itself is modeled by implementing a mechanistic model known as the general rate model (GRM), which is composed of a set of partial differential equations based on one-dimensional convective and dispersive mass transfer^15^. The equations describe the component concentrations in the interstitial mobile, stagnant particle, and solid mobile phase as functions of space and time. Various parameters can be set to reflect the column, particle, and resin-specific characteristics. The set of equations are solved numerically using a technique called orthogonal collocation. This procedure discretizes the spatial domain of the equations and converts them into a system of ordinary differential equations. The column equations and general system equations are combined and solved in the temporal domain using a backward Euler step method discretization technique using the simulation testbed for the liquid chromatography package. To speed up the simulation times, we use a simplified abstraction of what we refer to as spent media or impurities. This is a symbolic lumped concentration representing the substance that one would wish to separate from the product in this process. The tracked compounds in our system indexed by $i = 0,\dots ,3$, where 0 represents the concentration of salt, 1 represents the protein, 2 represents the impurities, and 3 represents the wash buffer.

The relationship between the flow rates and liquid volumes in parts of the system is governed by the following equation:

1 $$\frac{d{V}_{i}}{dt}={F}_{i}$$

where $V$ represents the volume of liquid contained in one of $i=1,\dots ,{N}_{\mathrm{v}}$, for ${N}_{\mathrm{v}}$ vessels, and ${F}_{\mathrm{i}}$ represents the specific flow rate for vessel $i$. All vessels upstream of the column are combined into the column inlet flow, ${F}_{\mathrm{in}}={\sum }_{i=1}^{3}{F}_{i}$.

To model the column, consider a spatial variable along the axis, $z\in {\Omega }_{z}=[0,L]$, where $L$ is the column length. Resin particles with radius ${r}_{\mathrm{p}}$ and radial axis, $r\in {\Omega }_{r}=\left[0,{r}_{\mathrm{p}}\right],$ are uniformly distributed along the column axis. To advance the model in time we let $t\in \left[0,{t}_{\mathrm{f}}\right],$ for some simulation time ${t}_{\mathrm{f}}$. The GRM used in this study describes convective mass transport and axial dispersion in the column volume using the following equation:

2 $$\frac{\partial {c}_{i}}{\partial t}=-u\frac{\partial {c}_{i}}{\partial z}+{D}_{\mathrm{ax},i}\frac{{\partial }^{2}{c}_{i}}{\partial {z}^{2}}-\frac{1-{\epsilon }_{\mathrm{c}}}{{\epsilon }_{\mathrm{c}}}\frac{3{k}_{\mathrm{f},i}}{{r}_{\mathrm{p}}} \left({c}_{i}-{c}_{\mathrm{p},i}\left(\cdot ,\cdot ,{r}_{\mathrm{p}}\right)\right)$$

The mobile phase concentration of component ${c}_{i}\left(t,z\right)$ is given for $i=0,\dots {,N}_{\mathrm{c}}$, for ${N}_{\mathrm{c}}$ components. The interstitial velocity $u$ is related to the flow rate by $u={F}_{\mathrm{in}}/{A}_{\mathrm{c}}{\upepsilon }_{\mathrm{c}}$, where ${A}_{\mathrm{c}}$ is the column cross section area and ${\epsilon }_{\mathrm{c}}$ the column porosity. Furthermore ${D}_{\mathrm{ax},i}$ is the axial dispersion coefficient and ${k}_{\mathrm{f},i}$ is the film mass transfer coefficient. The final term includes the mass flux into the pore phase, with the pore phase concentration denoted by ${c}_{\mathrm{p},i}(t,z,r)$. The mass balance in the pore phase is given by

3 $$\frac{\partial {c}_{\mathrm{p},i}}{\partial t}={D}_{\mathrm{p},i} \left(\frac{{\partial }^{2}{c}_{\mathrm{p},i}}{\partial {r}^{2}}+\frac{2}{r}\frac{\partial {c}_{\mathrm{p},i}}{\partial r}\right)-\frac{1-{\epsilon }_{\mathrm{p}}}{{\epsilon }_{\mathrm{p}}}\frac{\partial {q}_{i}}{\partial t}$$

where ${D}_{\mathrm{p},i}$, is the pore diffusion coefficient and ${\epsilon }_{\mathrm{p}}$ represents particle porosity. To represent binding interactions between solute components and stationary phase, we use the mobile phase modulator Langmuir model^16^. The model allows modulation of component adsorption and desorption rates by salt concentration. This dependency is evaluated for each individually modelled particle along the column axis. This interaction is governed by the following equation.

4 $$\frac{\partial {q}_{i}}{\partial t}={k}_{\mathrm{a},i}{e}^{\upgamma {c}_{\mathrm{p},0}}{c}_{\mathrm{p},i}{(q}_{\mathrm{max},i}-{q}_{i})-{k}_{\mathrm{d},i}{c}_{\mathrm{p},0}^{\upbeta }{q}_{i}$$

In the model ${k}_{\mathrm{a},i},{k}_{\mathrm{d},i},$ denote adsorption, desorption rate constants, respectively; ${q}_{\mathrm{max},i}$ represents the adsorption capacity; $\upgamma$ and $\upbeta$ modulation constants. In these equations, we only used β, to obtain the desired interaction in our system model by setting it to 1. The salt component in the system, ${c}_{\mathrm{p},0}, {q}_{0}$, is inert; thus $\frac{\partial {q}_{0}}{dt}=0$. The upstream mass flux is transported into the column via the inlet boundary condition:

5 $$u{c}_{\mathrm{inj},i}(t)=u{c}_{i}(t,0)-{D}_{\mathrm{ax},i}\frac{\partial {c}_{i}}{\partial z}(t,0)$$

where ${c}_{\mathrm{inj},i}$, defines the concentration of injected components. The outlet boundary is defined as follows.

6 $$\frac{\partial {c}_{i}}{\partial z}\left(t,L\right)= 0$$

Mass flux into the particles is represented by the following relation:

7 $${\epsilon }_{\mathrm{p}}{D}_{\mathrm{p},i}\frac{\partial {c}_{i}}{\partial {r}^{2}}\left(t,z,{r}_{\mathrm{p}}\right)={k}_{\mathrm{f},i} \left({c}_{i}(t,z)-{c}_{\mathrm{p},i}(t,z,{r}_{\mathrm{p}})\right)$$

Finally, spherical symmetry in the resin particles gives the following condition:

8 $$\frac{\partial {c}_{{p}_{i}}}{\partial r}\left(t,z,0\right)= 0$$

The equations are combined into a single semi-discrete system of equations with the spatially dependent derivatives discretized into ${N}_{z},N_r$ elements for *z* and *r* using orthogonal collocation^17^. Each variable is then approximated by interpolating polynomials of order ${N}_{\mathrm{p}}$ in each element with ${C}^{1}$-continuous boundaries. To solve the semi discrete system, a fixed-point iteration using the backward Euler method was applied:

9 $${y}_{k+1}={y}_{k}+hf\left({y}_{k+1},{t}_{k+1}\right).$$

Here, *h* indicates timestep length and *k* the discretization of the time domain for ${k=1,\dots ,N}_{t},$ for ${N}_{t}$ total timesteps. The semi-discrete system is merged into a single sparse matrix and solved using LU-factorization.

The simulated system exposes the control surfaces of the modeled physical system. This allows an operator to set the volumetric flow rates from the upstream vessels using the flow controller. The simulator calculates the flow rate into the column and the average flow rate of the phases migrating through the column. The column inlet concentrations are derived from the initial vessel concentration ${c}_{\mathrm{init},i}$ and flow rates in the flow controller ${c}_{\mathrm{in},i}=\frac{{F}_{i}{c}_{i}}{{F}_{\mathrm{in}}}$. The column simulation continuously calculates the concentration of the components in the column and, as the solution reaches the column outlet, the output valve allows the operator to direct the flow toward the product or waste vessels.

The simulation system observations are recorded in a time-indexed state vector. This vector maintains the current and historical records of component concentrations in the system vessels, component concentrations along the length of the chromatography column, component solution volumes in the system vessels, and the approximate component mass contained in the column and system vessels. The flow controller and output valve are controlled programmatically, with the flow controller able to set flows for each component between zero and 2.0 × 10^–6^ m^3^ s^−1^, and the output valve was set to either direct the flow to the waste or product tank.

### Column variability

To model the column variability, a set of test columns with varying performance was created. In these columns, the initiation parameters were adjusted to create unique simulation environments. Randomized parameters for the columns were generated by adding the product of the initial value scaled with a uniformly distributed error:

10 $$f\left(x\right)=x+x \cdot S \cdot X$$

Here, $X\sim U\left(-\mathrm{1,1}\right)$ is a uniformly distributed random variable, $S\in (\mathrm{0,1}]$ is a scaling variable, and *x* is some input parameter. In the experiment, the axial dispersion, radial dispersion, column porosity, and resin porosity were subjected to this treatment. The scaling term was maintained for all test columns, thereby incorporating the maximum relative deviation for each parameter (relevant initial parameters in Table 1). Environmental variation was achieved by sampling *X* and multiplying with the error term.

**Table 1:** Parameters used in simulation including randomized columns which are derived by perturbation.

Simulation parameter	Value
${c}_{0 }(\mathrm{mAb}, \mathrm{sm}, \mathrm{salt})$	$[\mathrm{0.1,1},1] \times {10}^{-7} \, \mathrm{ g}{\mathrm{m}}^{-3}$
${D}_{\mathrm{ax}}$	$1\times {10}^{-7} \, {\mathrm{m}}^{2}\, {\mathrm{s}}^{-1}$
${D}_{\mathrm{p}}$	$1.2\times {10}^{-6} \, {\mathrm{m}}^{2} \, {\mathrm{s}}^{-1}$
${k}_{\mathrm{f}}$	$8.0\times {10}^{-3}$
${k}_{\mathrm{a}}(\mathrm{mAb}, \mathrm{sm}, \mathrm{salt})$	[1.0,0.0,0.0] ${\mathrm{s}}^{-1}$
${k}_{\mathrm{d}}(\mathrm{mAb}, \mathrm{sm}, \mathrm{salt})$	[0.01,0,0] ${\mathrm{s}}^{-1}$
${q}_{\mathrm{max}}(\mathrm{mAb}, \mathrm{sm}, \mathrm{salt})$	[1,0,0] ${\mathrm{gm}}^{-3}$
${r}_{\mathrm{p}}$	$42.5\times {10}^{-5} \, {\mathrm{m}}$
${\upepsilon }_{\mathrm{c}}$	0.4
${\upepsilon }_{\mathrm{p}}$	0.333
$L$	0.197 $\mathrm{m}$
${A}_{\mathrm{c}}$	$7.85\times {10}^{-5} \, {\mathrm{m}}^{2}$
$h$	15 $\mathrm{s}$
${N}_{z}$	40
${N}_{r}$	5

### Deep reinforcement learning

**Figure 2:**
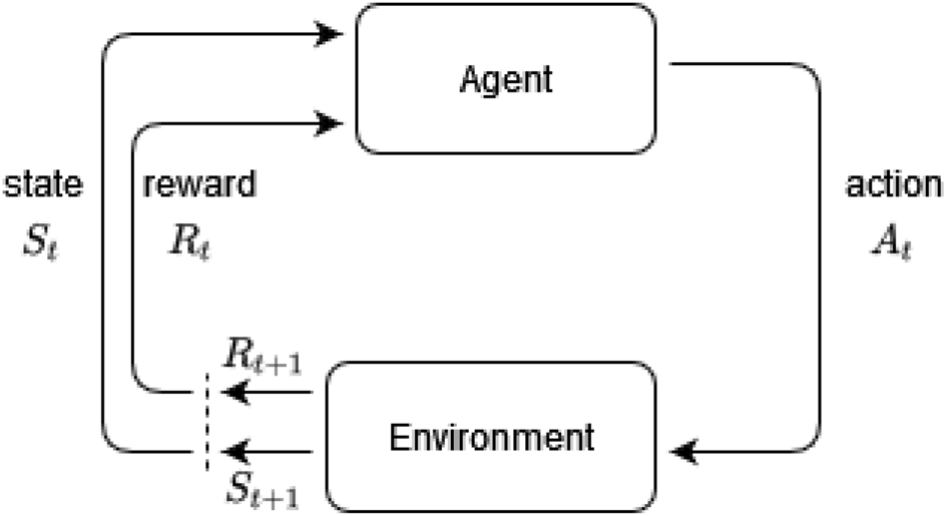
Illustration of reinforcement learning agents training by interacting with an environment. For each state *S*_*t*_, the agent generates an action *A*_*t*,_ which produces an action and reward for time *t* + 1.

Deep RL combines the classical field of reinforcement learning with deep artificial neural networks^18^ to optimize the behavior of an agent in an environment using machine learning algorithms. The agent is optimized by maximizing a reward function, which incentivizes the desired behavior and penalizes the undesired behavior. During RL, the environment starts in an initial state *S*_*t*_ and the agent selects an action A_*t*_; the environment is then updated based on the action, and it returns the next state *S*_*t*+1_ and a corresponding reward associated with that state *R*_*t*+1_ (see Fig. 2). The agent, expressed as an artificial neural network (ANN), is continuously updated by adjusting the ANN weights using the backpropagation algorithm. The interaction between the agent and environment is continued until the environment reaches a terminal state or for a set number of iterations. For the agent to learn an optimal policy, one must run multiple episodes of interactions with the environment from start to finish, often referred to as episodes or trajectories.

**Figure 3:**
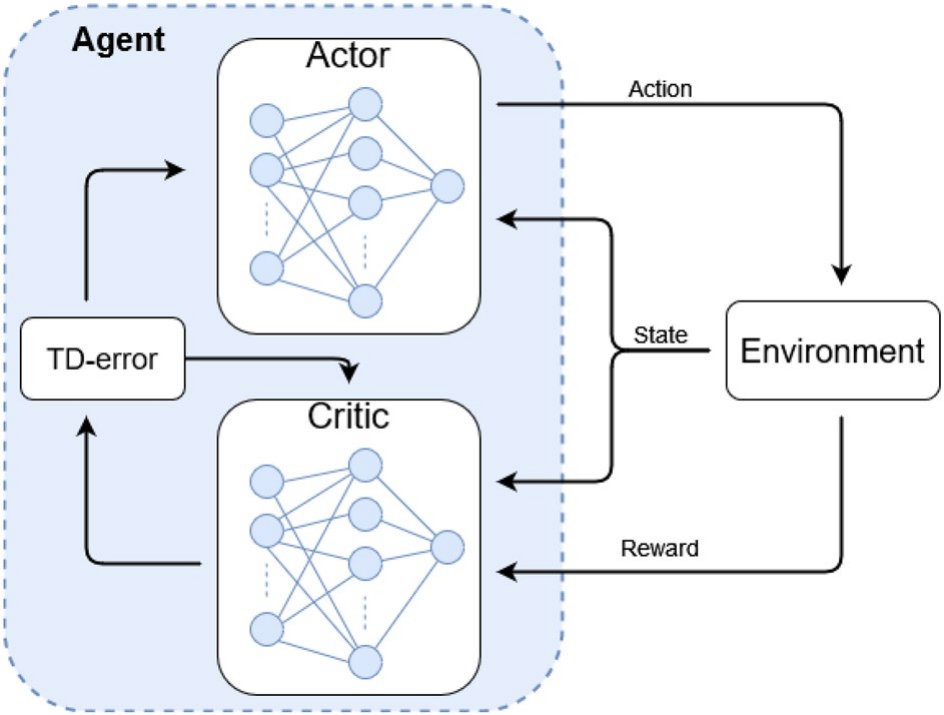
Actor–critic agent interaction with an environment. The actor part of the network predicts subsequent actions while the critic estimates the reward gained by the action–state pair and is used to train the actor network.

Here, we used a deep RL algorithm called twin-delayed Deep Deterministic Policy Gradient-method (TD3)^19^ which is an extension of the widely used DDPG^20^. TD3 is an actor–critic method (visualized in Fig. 3), meaning that the agent has two components during training: an actor and a critic, both implemented as ANNs. The actor network takes a state S_*t*_ and predicts the next action A_*t*_, which is also referred to as the agent’s policy. On the other hand, the critic network is tasked with estimating the future accumulated reward gained by a state–action pair and is used to train the actor to produce actions that yield high rewards based on the current state. TD3 is a temporal difference (TD) learning algorithm^21^, which means that it bootstraps the estimate of future rewards using a value function instead of running the interaction with the environment until the end and gathering all future rewards. The value function is estimated by the critic network, which iteratively gets better at making these estimations. To train the critic network, we estimate how well it performs in a bootstrapping fashion by calculating $T{D}_{\mathrm{error}}$, defined as:

11 $$T{D}_{\mathrm{error}}= {R}_{t}+ \upgamma Q\left({S}_{t+1},{A}_{t+1},{w}_{\mathrm{Q}}\right)-Q\left({S}_{t},{A}_{t},{w}_{\mathrm{Q}}\right)$$

where *R*_*t*_ *S*_*t*_, and *A*_*t*_, are the reward, state, and action of current timestep *t*, and *S*_*t*+1_*, A*_*t*+1_*,* are the state and action of timestep *t* + 1.* Q* is the critic network with weights ${w}_{\mathrm{Q}}$ and is used to estimate the future rewards; also referred to as the *Q*-value, for a state–action pair. The discount factor γ is used to weigh the rewards of far-future actions to indicate that they are more difficult to predict than near-future actions. $T{D}_{\mathrm{error}}$ approaches zero when the networks improve because the *Q*-value (accumulated future rewards) of timestep *t* should be equal to the reward from timestep *t* added to the *Q*-value of timestep *t* + 1 given a perfect *Q*-value estimation. Given:

12 $$Q\left({S}_{t},{A}_{t},{w}_{\mathrm{Q}}\right)= {R}_{t}+\upgamma Q\left({S}_{t+1},{A}_{t+1},{w}_{\mathrm{Q}}\right)$$

$T{D}_{\mathrm{error}}$ is used as the loss/cost-function to train the critic with standard deep learning optimization techniques such as gradient decent. The actor is trained to produce the optimal policy using the estimated Q-value $\widehat{R}$ from the critic network:

13 $$\widehat{R}=Q\left({S}_{t},{A}_{t},{w}_{\mathrm{Q}}\right)$$

where action ${A}_{t}$ is the product of the action networks $\uppi$ policy based on state ${S}_{t}$ and weights ${w}_{\uppi }$ as

14 $${A}_{t}=\uppi \left({S}_{t},{w}_{\uppi }\right)$$

Being implemented as an ANN, the critic’s reward estimate is differentiable, meaning that it can be used to optimize the actor by using gradient ascent to update the actors’ network weights ${w}_{\uppi }$ and yield a better policy. In practice, this is achieved by freezing the network weights of the critic and using a gradient descent on the negative future reward $\widehat{R}$ estimated for the current action–state pair. The actor’s loss function is then given by

15 $${L}_{\mathrm{actor}}=-Q\left({S}_{t},\uppi \left({S}_{t},{w}_{\uppi }\right),{w}_{\mathrm{Q}}\right)$$

By leveraging bootstrapping in TD-learning, only the state, action, reward, next state, and next action for a timestep *t* are needed to calculate all the losses to train an RL agent. This is often referred to as state–action–reward–state–action (SARSA), which means that when the agent interacts with the environment, it is possible to save SARSA tuples for each time point and use the experiences for training^22^.

When training the agent, we run the simulation (or real-world interaction) with a specified number of iterations or until a terminal condition is reached. During training, SARSA experiences are recorded and put into a memory buffer, and after a predefined number of steps in the environment, the agent will sample the memory buffer for a fixed number of times and train the agent networks with those experiences. In practice, this means that the training examples drawn can originate from both current and past episodes. This makes training more stable because drawn experiences are less correlated with each other compared to chronologically following ones^23^.

In our experiments, we used a memory buffer to run multiple experiments in parallel and gathered them in a single memory buffer. To allow the agent to explore new possible actions, random noise was added to the actions. The noise amplitude decayed over time to incentivize exploration early on and gradually transferred to a higher degree of exploitation of the environment. By combining multiparallel training and action noise, the same actor explores different actions in the same environment and state owing to noise, which improves learning because of to its regularizing effect.

### Reward function

A critical aspect of RL is the definition of a suitable reward function. In this experiment, the total reward for each state was calculated and then subtracted from the previously recorded rewards to give us a reward that reflects the changes made on a step-by-step basis as follows:

16 $${\Delta }_{R} ={R}_{t}-{R}_{t-1}$$

where ${R}_{t}$ is the reward at time *t*, and ${R}_{t-1}$ is the reward at time *t* − 1.

In our chromatography use case, the reward function must consider multiple factors. First, the product is separated into the product tank and waste into the waste tank (see Fig. 1). This is reflected in the following terms:

17 $${\Delta }_{\mathrm{mab}} =\mathrm{mab}_{\mathrm{prod}}-\mathrm{mab}_{\mathrm{waste}},$$

18 $${\Delta }_{\mathrm{sm}} =\mathrm{sm}_{\mathrm{waste}}-\mathrm{sm}_{\mathrm{prod}},$$

where ${\Delta }_{\mathrm{mab}}$ is the mass of mAb collected in the product tank $\mathrm{mab}_{\mathrm{prod}}$ minus the mass that ends up in the waste tank $\mathrm{mab}_{\mathrm{waste}}$. Inversely, the waste product ${\Delta }_{\mathrm{sm}}$ is the mass of waste in the waste tank $\mathrm{sm}_{\mathrm{waste}}$ subtracted by the mass of waste in the product tank $\mathrm{sm}_{\mathrm{prod}}$. We then define the mass-reward function as follows:

19 $${R}_{\mathrm{mass}} = {(\alpha \cdot \Delta }_{\mathrm{mab}} +{\Delta }_{\mathrm{sm}} )$$

where ${\Delta }_{\mathrm{mab}}$ is multiplied by a real-valued scaling factor $\alpha$ to reflect the ratio between the waste and mAb ($\alpha =5$ in our experiments). Preliminary experiments showed that training with $\alpha =1$ made the agent ignore mAb in favor of putting all the waste in the waste tank.

Further preliminary experiments showed that training the algorithm using *R*_mass_ resulted in an agent that maximized the mass difference to achieve a higher reward than perfect separation but with very low mAb purity. Perfect separation requires that less feed (total amount of processed medium) is placed into the system after loading, but *R*_mass_ can be maximized by simply keeping the feed high to accumulate more product. To improve purity, we defined an additional reward function to incentivize a higher concentration of the resulting product:

20 $${R}_{\mathrm{concentration}}=\mathrm{max}\left( \frac{\mathrm{mab}_{\mathrm{prod}}}{\mathrm{mab}_{\mathrm{prod}}+\mathrm{sm}_{\mathrm{prod}}} , x\right)$$

using minimum value of *x* = 0.1; however, it would increase as the purity of mAb in the product tank increases. This yields the following intermediary reward function:

21 $${R}_{1} = {R}_{\mathrm{mass}}\cdot {R}_{\mathrm{concentration}}$$

Optimizing ${R}_{1}$, the agent puts all products in the column and never washes them out to only accumulate reward from waste correctly placed in the waste tank, leaving an empty product tank. Therefore, we introduced a penalty for mAb left in the column tank as follows:

22 $${P}_{\mathrm{remaining}}= \left\{\begin{array}{ll}{\mathrm{mab}}_{\mathrm{col}} & \text{if }{\mathrm{mab}}_{\mathrm{col}}>{\mathrm{mab}}_{\mathrm{max}}\\ 0 & \text{otherwise}\end{array}\right.$$

where $\mathrm{mab}_\mathrm{col}$ is the mAb in the column and ${\mathrm{mab}}_{\mathrm{max}}$ is a threshold value that determines if there is too much mAb in the column at the end of the run. Further, we added another reward to incentivize “clean” mAb in the product tank.

23 $${R}_{\mathrm{clean}}=\mathrm{max}\left(\mathrm{mab}_{\mathrm{prod}}-\mathrm{sm}_{\mathrm{prod}},0\right)$$

Combining the terms yields the second intermediary reward function:

24 $${R}_{2} =\beta \cdot \left({R}_{\mathrm{mass}}\cdot {R}_{\mathrm{concentration}}+{R}_{\mathrm{clean}} - {P}_{\mathrm{remaining}}\cdot {I}_{\mathrm{done}}\right)$$

where the indicator ${I}_{\mathrm{done}}$ is 1 if the simulation has finished and 0 otherwise, and $\beta$ is a real-valued scaling factor ($\beta ={10}^{6}$ in our experiments).

Using ${R}_{2}$, the agent consistently achieved a product purity of approximately 35% but struggled to improve beyond that. To incentivize increased purity, we added a reward that increases linearly based solely on purity, ignoring the amount of product, after achieving 30% concentration:

25 $${R}_{\mathrm{purity}} = \mathrm{max}\left(\frac{{R}_{\mathrm{concentration}}-0.3}{0.7}, 0\right)$$

To define our final reward function:

26 $$R=\beta \cdot \left({R}_{\mathrm{mass}}\cdot {R}_{\mathrm{concentration}}+{R}_{\mathrm{clean}}+\gamma \cdot {R}_{\mathrm{purity}} - {P}_{\mathrm{remaining}}\cdot {I}_{\mathrm{done}}\right)$$

where γ is a real-valued scaling factor that adjusts the importance of purity at later stages during training (γ = 100 in the experiments).

## Experiments

The environment simulated a chromatography process running 3000 s with the agent interacting with the environment every 15 s, totaling 200 timesteps per episode. In the running simulation, the agent sets the volumetric flow rates from the upstream vessels using the flow controller. Simultaneously, as the fluid flows through the column outlet, the agent can direct flow toward the product or waste vessels. In the experiment, both the actor and critic observed the following parameters: wash flow rate, mass of components fed into column inlet, mass of components leaving column outlet, and approximate mass of components currently in column. The observation data were approximated using the known initial concentrations and flow rates together with the simulated concentrations in the column outlets.

During training, the column parameters were randomly initiated for each new episode, according to the method described in the Column Variability section. A scaling factor *S* of 0.2 was used for training. To generate a hold-out set for validation, eight test columns were generated using a random initiation scaling factor of 0.05. In all cases, the environment state representations for 10 consecutive timesteps were concatenated to create an input time sequence. Furthermore, all the observed states for the agent were normalized to the range of [0,1]. The scaling factors were determined by experimentally running the chromatography simulator, identifying the highest values of the different measurements, and then dividing the state vector by the maximum values.

**Figure 4:**
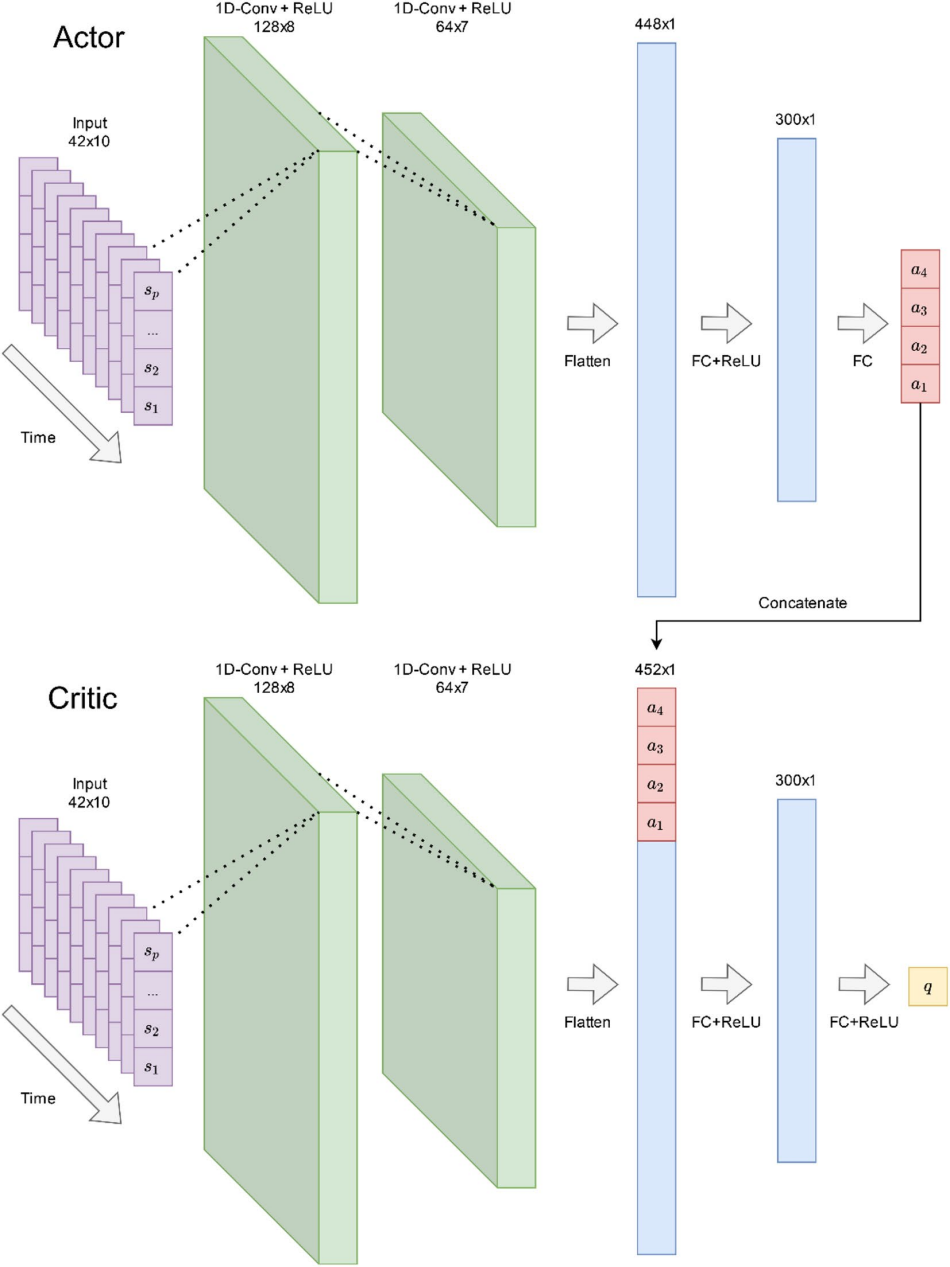
Network architecture diagrams of the actor- and critic-temporal convolutional networks.

Both critic and actor networks used a three-layer temporal convolutional network (TCN) architecture, which is common for processing time-series data^24^ and is illustrated in Fig. 4 The input was passed into two 1D-convolutional layers with 128 and 64 kernel filters, kernel size of 3, and stride of 1, each followed by rectified linear unit (ReLU) activation before flattening the time dimension. The actor’s flattened vector was passed into a fully connected layer comprising 300 neurons, followed by ReLU activation^25^ before passing into the last fully connected layer mapping to the four-dimensional output representing the actions that can be taken in the simulated chromatography device. The critic’s flattened vector was concatenated with the actor’s output action vector before performing the same transform as the actor, mapping to a single output representing the *Q*-value (estimated future rewards) based on the current state–action pair.

Both actor- and critic-TCNs were trained using stochastic gradient descent using the ADAM^20^ and a learning rate of 4⋅10^–4^. A priority-experience memory buffer was used to store historical experiences in the form of state, action, reward, next state, and next action for timestep *t* (SARSA), tuples with a buffer size of 10^–6^. During each iteration, 200 SARSA tuples were sampled from the memory buffer four times for every 4 interactions with the environment to calculate the policy and *Q*-value losses and update the actor- and critic-TCNs. Following TD3^19^, we updated the critic twice for every actor update. The best model was saved for each run and used to plot and evaluate performance.

## Results

**Figure 5:**
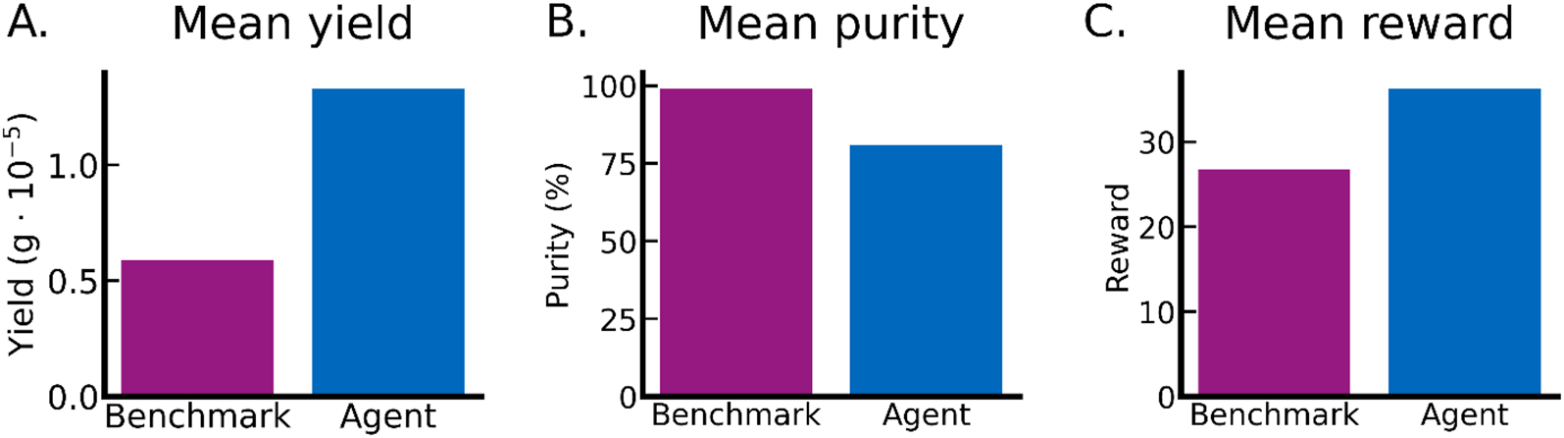
Comparison of agent generated and benchmark process in term of total yield, product purity, and reward. The agent values are average results from the predefined columns with noisy parameters.

When benchmarking our RL agent against an expert human-designed chromatography recipe on the hold-out test set columns, the RL agent achieved higher productivity but lower purity (Table 2). In terms of the total reward, the RL agent outperformed the human benchmark (Fig. 5C). This is also the case for mass of captured mAb, 1.33⋅10^–5^ g for RL agent compared to 0.59⋅10^–5^ g for human benchmark, translating to a 125% productivity increase (Fig. 5A). However, the increased productivity comes at the cost of lower purity, where the human benchmark achieved 99% purity compared to the 81% purity of the RL agent, translating to 18 pp lower purity (Fig. 5B).

**Table 2:** Comparison of an agent trained in an environment using column with random initializations against a human made benchmark policy over a pre-defined test-set of 8 columns.

	Agent (mean random)	Benchmark (mean random)
Reward score	36.29	26.78
mAb prod (1e^−5^ g)	1.33	0.59
sm prod (1e^−5^ g)	0.32	0.0052
mAb waste (1e^−5^ g)	0.15	0.073
Purity %	81.01	99.12
Recovered cycled mAb %	89.63	88.94
Recovered total mAb %	66.3	29.61

Score is a value calculated with the defined reward function for the agent training, we also show the amount of mAb captured in the product and waste vessels as well as the purity of mAb in the product vessel, ${c}_{\mathrm{mAb}}/\left({c}_{\mathrm{sm}}+{c}_{\mathrm{mAb}}\right)$. Recovered cycled mAb indicates how much of the target protein was recovered in the product vessel after cycling the feed stock. Recovered total mAb indicates how much target protein was recovered in relation to initial target protein mass in the feed vessel.

**Figure 6:**
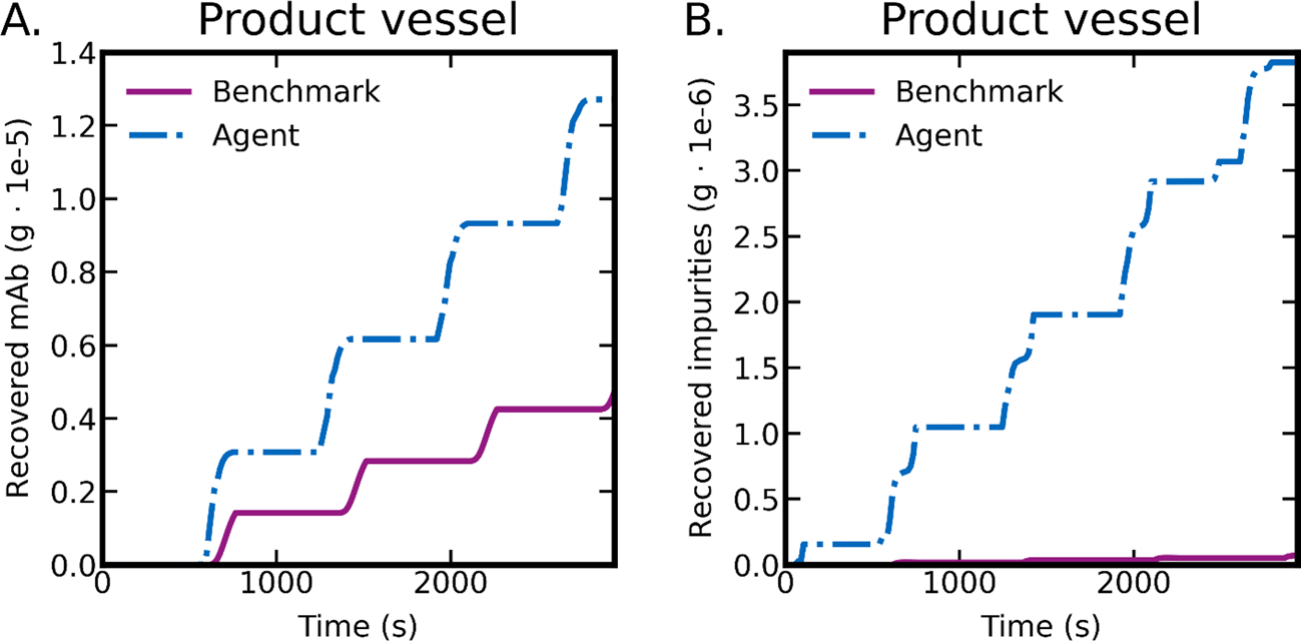
Comparison of agent and benchmark performance in terms of component mass in product vessel. Here, (**A**) shows the mass of mAb recovered from the separation process and (**B**) shows impurities which have failed to be separated into the waste vessel.

**Figure 7:**
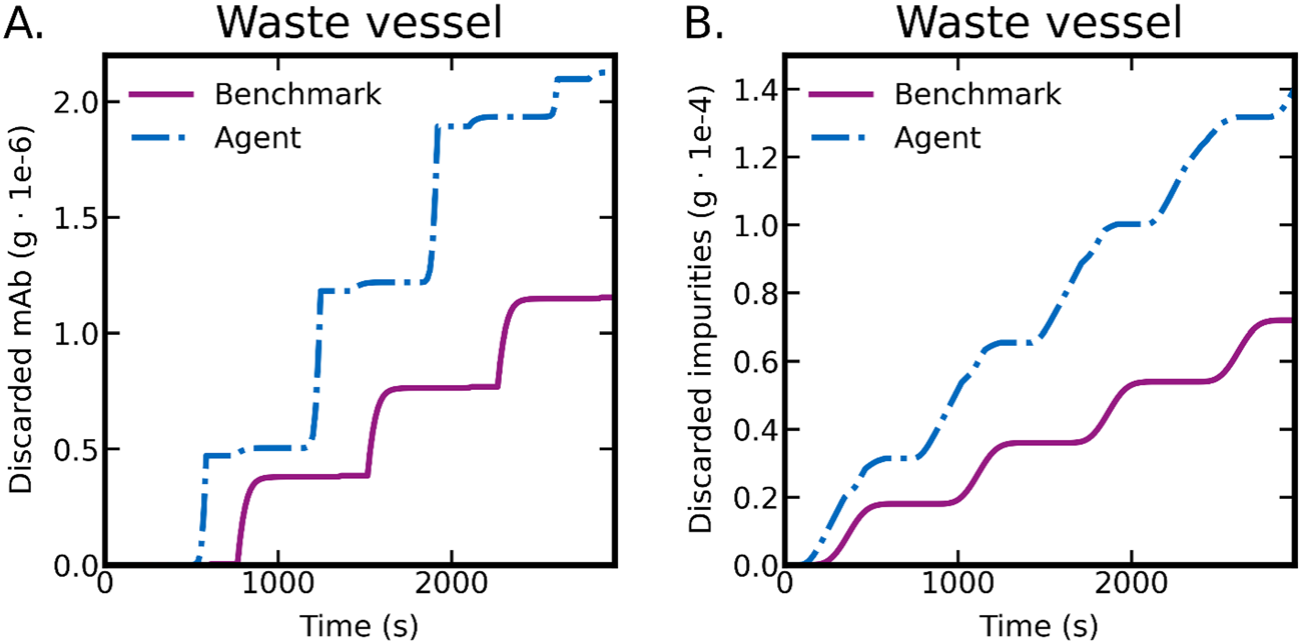
Comparison of agent and benchmark performance in terms of component mass in waste vessel. Here, (**A**) shows the mass of mAb discarded during separation process and (**B**) shows impurities which have correctly been separated into the waste vessel.

To provide insight into the agent’s learned chromatography recipe, we visualize its performance over an illustrative batch process in Figs. 6 and 7. The figures display a comparison of resulting outputs generated by the agent-derived control policy and the human designed benchmark policy. The RL agent achieved an expected step-wise separation of mAb (Fig. 6A), but doesn’t achieve the same degree of purity as the benchmark policy (Fig. 6B). In Fig. 7A it is evident the agent also tends to waste more product than the benchmark policy.

**Figure 8:**
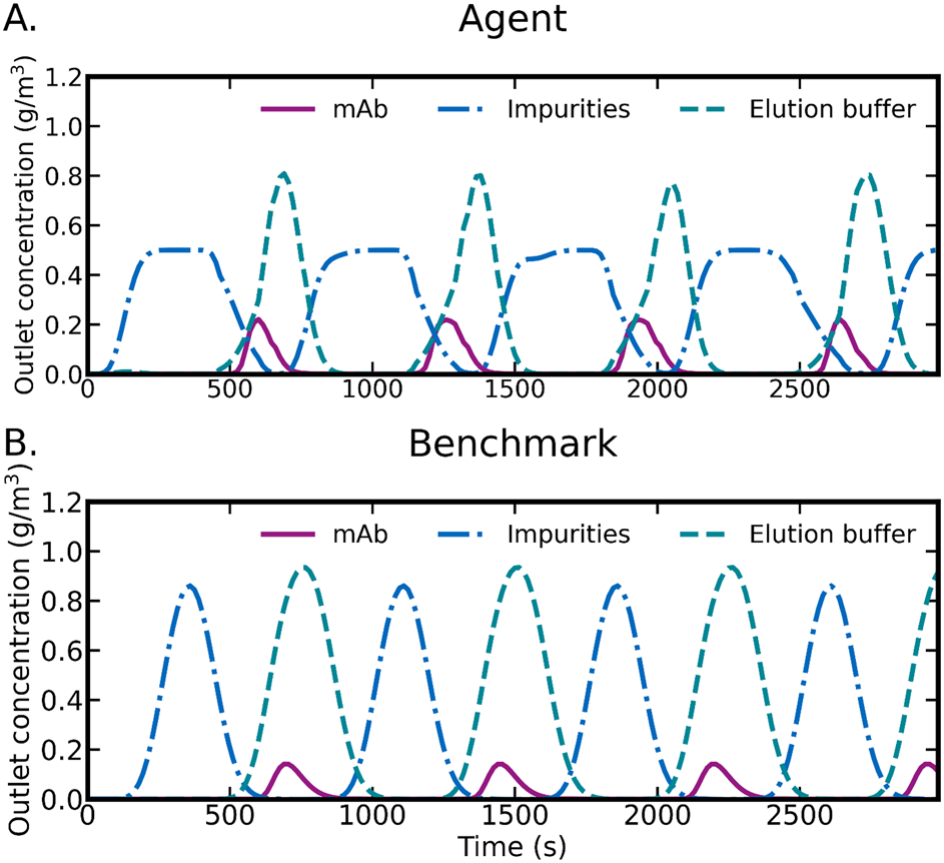
Comparison of agent (**A**) and benchmark (**B**) performance in terms of column outlet concentrations. In each figure, concentrations of mAb, impurities, and elute-buffer are visualized.

**Figure 9:**
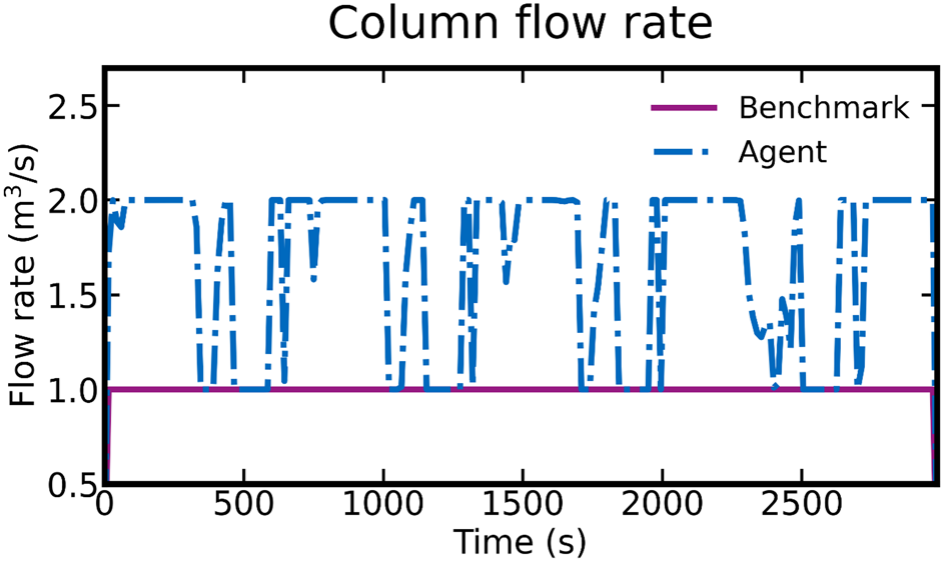
Comparison of agent and benchmark control inputs in terms of total column flow rates. Here, the agent generated control policy pumps several species simultaneously thereby increasing the total flow rate through the column.

Inspecting the control actions by visualizing the outlet concentrations of impurities and product flowing out of the column (Fig. 8) shows that the trained agent converges to a well-behaved cyclical pattern (Fig. 8A) similar to the human benchmark (Fig. 8B). The RL agent successfully adapted to the column at hand and concluded four load–wash–elute cycles within the designated time frame, whereas the fixed human benchmark policy ended in the middle of a cycle. We also note that the RL agent decreased cycle time by combining the wash and feed flows in an unexpected manner (Fig. 9). This shows that our agent is capable of adaptively using the provided control interfaces to improve productivity when compared to a standard constant flow rate process.

## Discussion

Conventional approaches to chromatography control have focused on determining the binding capacity of a chromatography column and using this information to inform the optimal control policy. The concept of column binding capacity is a common simplification for understanding the performance of chromatography systems based on the idea that there is a theoretical maximum amount of product that can be captured by the column, and the control objective is to approach that limit. Unlike previous works, we did not rely on the concept of binding capacity. Instead, we focused on the overall efficiency of the system (such as the total product captured in the product collection vessel compared to the impurities directed to the waste vessel). Consequently, our approach is more broadly adaptable and does not depend on the assumed parameters of the column. Even if this experiment is performed in a single-column setup, we hypothesize that the methodology can be transferred to a multi-column and continuous process context, where it might be even more relevant because more factors need to be taken into consideration—we are keen to see further exploration in this direction.

Current approaches to controlling resin liquid chromatography systems use fixed policies derived from experimental breakthrough curves. However, such fixed policies fail to account for column variability and capacity decreases due to usage, leading to suboptimal performance. Considering that there is considerable column variability due to randomness during, for instance, column packing combined with the high cost of chromatography resin, there is a great incentive to develop more adaptable strategies. RL has been used to develop adaptable control policies in other domains, and existing applications in chromatography impose strict limits on the available action^11^. We believe that by using these methods, one can derive a method that can adapt to the variability between columns and during runs.

We show that our RL agent successfully learns how to generate a control policy by interacting with a simulated chromatography device without any prior knowledge, going from completely random actions to a complex control scheme. Furthermore, by providing the RL agent freedom in the control actions available and training it on variable columns, it learns to adapt the column at hand and achieves better performance than a human-designed benchmark policy. We believe that this is the first proof-of-concept showing that RL can be used to develop new policies that are adaptable to the real-world variability of chromatography columns. This is important because control policies are currently designed per column, which incurs a significant amount of extra work. We demonstrate that it is feasible to use a single policy learned by an RL agent across a wide panel of chromatography columns, removing the need for column-specific calibration. In particular, the RL agent only observes sensor readings from outside the column, meaning that it can be used using current process-analytical technologies.

However, we emphasize that both improvement and validation are required before any commercial application. For instance, our RL agent did not achieve as clean mAb as the human-designed benchmark policy, even though it achieved a higher reward score by collecting more products in a fixed amount of time. Although we put significant effort into designing a suitable reward function, we believe that it needs further adjustments to improve purity to be on par with expert policies with higher total productivity. We are also confident that more extensive hyperparameter optimization will help improve the total reward and product purity.

We acknowledge that, despite significant effort, our reward function may require further refinement to increase product purity and align with expert policies yielding higher total productivity. We are confident that optimizing both the hyperparameters related to the algorithm, such as the learning rate and batch size, and those related to the reward function can lead to improvements in total reward and product purity.

Currently, the $\alpha$ parameter balances the reward given for correctly sorting waste and product, reflecting the fivefold difference in their quantities. The $\gamma$ parameter, defining the importance of purity, has shown stable training with the proposed values, though an extensive search for a potentially better value has not been conducted. The $\beta$ term is a reward scaling factor that provided satisfactory results while maintaining the same learning rate for the actor and critic. Further adjustments or removal of the term might be possible with changes to the critic's learning rate and the scaling of other reward parameters.

Our reward function design, which stemmed from iterative adjustments based on the observed agent behavior during preliminary experiments, incorporated dense and shaped reward elements. The $\Delta R$ calculation, a form of dense reward, provides the agent with immediate feedback at each timestep, essential given the complexity of the task. Shaped rewards encapsulate multiple significant factors pertinent to the chromatography process, including aspects such as the mass and concentration of the product and waste, penalties for undesirable outcomes like residual mAb in the column, and incentives for product purity. This comprehensive structure effectively guides the agent towards desirable behavior and away from counterproductive actions.

Our results indicate the agent is tolerant to smaller changes in physical parameters: axial dispersion, radial dispersion, column porosity, and resin porosity. However, we anticipate the need to adjust the reward function and hyperparameters for significant changes in process conditions. This could include changes in material not captured by the current simulation environment or configurations of separation system. We also recognize that other types of reward functions might yield better results, and encourage such exploration in follow-up work.

The choice of the TD3 algorithm is motivated by its ability to address the common issue of overestimation biases^19^, through the introduction of twin Q-functions. Additionally, TD3 ensures more stable training due to delayed policy updates, which reduces the correlation between policy and value function updates. The algorithm also benefits from the regularization effects of target policy smoothing. While we propose comparisons of different algorithms as potential future work, to better determine what works best in this particular domain, we argue that TD3 is sufficient to demonstrate the feasibility of adaptive control based on deep reinforcement learning.

In terms of validation of our results, one shortcoming of this study is that it is limited to simulation, and it is likely that the simulator-trained agent is not directly transferrable to real-world use. However, domain randomization is a common technique to bridge transfer from simulation to real-world experimentation in RL for robotics^26^. The strength of this study is that it demonstrates the RL agent’s adaptability to variable columns, and we leave experimental studies for future work. Further, reducing the time required for training by, for instance, starting with imitation learning^27^ on a human-designed policy before letting the RL agent explore has also been left for a future work.

In terms of the actual policies learned by the RL agent, we would like to highlight the cyclical behavior the agent learns without any explicit supervision to do so. The learned cyclical behavior shares many similarities with the phases used in human-designed chromatography policies. When the agent loads the column with feed, the column will leak when it comes close to the column binding capacity and passing past this capacity will result in lower productivity and hence lower reward. Even though there are no hard-coded constraints disallowing the agent to overflow the column, the reward function penalizes it, and agent training will remove such behavior in search of maximizing the reward function. We would also like to highlight that given the freedom provided to the RL agent in terms of available actions, it may learn unexpected policies. In our case, the agent learns to shorten the cycle times by combining the wash- and feed flows. Such unexpected actions may serve as an inspiration for improving human-designed policies after suitable experimental validation.

## Data Availability

STLC is available at: https://github.com/sartorius-research/STLC.
